# EHMTI-0066. Effect of intrauterine growth restriction on the development of migraine and tension-type headache: the Nord-Trøndelag Health Study (HUNT-3)

**DOI:** 10.1186/1129-2377-15-S1-B40

**Published:** 2014-09-18

**Authors:** BS Winsvold, LM Jacobsen, LM Pedersen, JA Zwart

**Affiliations:** 1 Oslo University Hospital and University of Oslo, FORMI and Department of Neurology, Oslo, Norway; 2 Oslo University Hospital, Formi, Oslo, Norway

## Introduction

Little is known about the importance of intrauterine factors on the later development of headache.

## Aims

We aimed to study whether intrauterine growth restriction is associated with development of migraine and tension-type headache in young adulthood.

## Methods

We analysed data from 6,321 Norwegian adults age 19-41 years who participated in the Nord-Trøndelag Health Study (HUNT-3), using linked data on birth weight and gestational age from the Medical Birth Registry of Norway. Based on national reference values, participants were categorised as being born appropriate for gestational age (10th-90th percentile), small for gestational age (3rd-10th percentile) or very small for gestational age (<3rd percentile). The effect of intrauterine growth restriction on the presence of migraine or tension-type headache in young adulthood was analysed using logistic regression, adjusted for age and sex.

## Results

Compared with those born with a birth weight appropriate for gestational age, children born small or very small for gestational age had an increased risk for developing migraine (OR=1.23, 95% CI=0.98-1.55, and OR=1.69, 95% CI=1.18-2.41 respectively, p for trend=0.001), but not tension-type headache (OR=1.09, 95% CI=0.86-1.38 and OR=1.45, 95% CI=1.00-2.09 respectively, p for trend=0.053). When stratified by aura status, there was a significant association between intrauterine growth restriction and both of migraine with aura (p=0.011) and migraine without aura (p=0.019).

## Conclusions

Intrauterine growth restriction is associated with an increased risk for developing migraine in young adulthood. This suggests that migraine is caused partly by influences in early development.

No conflict of interest.

